# Emerging functions of m6A-modified circRNAs and their targeting strategies in lung cancer

**DOI:** 10.3389/fcell.2026.1794138

**Published:** 2026-04-17

**Authors:** Haipeng Wang, Yixiao Yuan, Chongxin Li, Xiulin Jiang, Jin Wang

**Affiliations:** 1 Hunan Provincial Key Laboratory of Regional Hereditary Birth Defects Prevention and Control, Changsha Hospital for Maternal and Child Care, Hunan Normal University, Changsha, Hunan, China; 2 The First Affiliated Hospital of Chongqing Medical University, Chongqing, China; 3 Department of Oncology, Qujing Central Hospital of Yunnan Province, Qujing, Yunan, China; 4 College of Life Science, University of Chinese Academy of Sciences, Beijing, China

**Keywords:** cancer stem cells (CSCs), circular RNA, ferroptosis, lung cancer, m6A modification, metabolic reprogramming, therapeutic resistance, tumor immune microenvironment

## Abstract

Lung cancer, especially non-small cell lung cancer (NSCLC), remains a leading cause of cancer-related morbidity and mortality worldwide, largely due to challenges in early diagnosis, pronounced tumor heterogeneity, and frequent therapeutic resistance. N6-methyladenosine (m6A), the most prevalent RNA epigenetic modification, participates in tumorigenesis and progression by regulating various aspects of RNA metabolism. Circular RNAs (circRNAs), characterized by their covalently closed loop structure and high stability, have emerged as important regulators of tumor proliferation, metastasis, and drug resistance. Increasing evidence indicates that m6A modification influences circRNA biogenesis, stability, and translational potential, while circRNAs can reciprocally modulate the m6A machinery or act as molecular scaffolds within m6A regulatory networks. In this mini-review, we summarize recent advances regarding m6A-modified circRNAs in lung cancer, with particular emphasis on their roles in tumor growth and metastasis, ferroptosis, cancer stem cell maintenance, resistance to radiotherapy, chemotherapy, and targeted therapy, metabolic reprogramming, and the tumor immune microenvironment. Additionally, we discuss the diagnostic and therapeutic potential of m6A-circRNA interactions. Finally, we highlight the need for future studies to elucidate the dynamic regulation and clinical translation of the m6A–circRNA axis, aiming to provide novel strategies for precision therapy in lung cancer.

## 1 Introduction

Lung cancer is one of the most common malignancies worldwide in terms of both incidence and mortality (Yuan et al., 2025). NSCLC accounts for approximately 85% of all lung cancer cases. Despite significant advances in targeted therapies and immunotherapy over the past decade, the 5-year survival rate of NSCLC remains unsatisfactory, largely due to difficulties in early diagnosis, pronounced tumor heterogeneity, and frequent development of therapeutic resistance (Le et al., 2021). The pathogenesis and progression of lung cancer involve a complex interplay of genetic alterations, epigenetic regulation, metabolic reprogramming, and remodeling of the tumor immune microenvironment (Romero, 2019). Among these mechanisms, RNA epigenetic modifications represent a crucial regulatory layer that influences RNA splicing, stability, transport, and translation, thereby profoundly affecting tumor biology (Gui and Yuan, 2021).

m6A is the most prevalent and dynamic reversible modification in eukaryotic mRNAs and non-coding RNAs. It is catalyzed by “writers” (e.g., METTL3, METTL14, WTAP) (Pajak et al., 2019; Jia et al., 2011; Liu et al., 2014; Zheng et al., 2013), removed by “erasers” (e.g., FTO, ALKBH5), and recognized by “readers” (e.g., YTHDFs, IGF2BPs, YTHDCs) (He et al., 2017; Xiao et al., 2016). m6A modification occurs across multiple RNA species, including messenger RNAs (mRNAs) (Wang Y. et al., 2024), long noncoding RNAs (lncRNAs) (Fang et al., 2022), microRNAs (pri-miRNAs) (Zhang et al., 2024), circular RNAs (circRNAs) (Du et al., 2022), and other noncoding RNAs (Wei et al., 2022). Transcriptome-wide mapping studies have shown that m6A is enriched near stop codons, within 3′untranslated regions (3′UTRs), and in long internal exons, typically occurring within the consensus RRACH motif (Wei et al., 2022). Accumulating evidence has demonstrated that m6A modification regulates diverse biological processes, including tumor cell proliferation, migration, metabolism, stemness maintenance, and immune evasion, and is closely associated with lung cancer development and treatment resistance (Hou et al., 2021). Circular RNAs (circRNAs) are a class of covalently closed RNA molecules generated through back-splicing of precursor mRNAs (Li et al., 2018). Unlike linear RNAs, circRNAs lack 5′caps and 3′poly(A) tails, which renders them resistant to exonuclease-mediated degradation and contributes to their high stability (Li et al., 2018). Importantly, circRNAs often exhibit cell type–specific and tissue-specific expression patterns, a feature primarily determined by the transcriptional activity of their host genes, alternative splicing regulation, and the involvement of specific RNA-binding proteins, rather than by their circular structure (Li et al., 2018). In addition to their structural features and stability, circRNAs exert diverse regulatory functions that are highly relevant to cancer biology. One of the most extensively studied mechanisms is their role in competing endogenous RNA (ceRNA) networks, in which circRNAs act as microRNA (miRNA) sponges to modulate the availability of miRNAs and thereby influence downstream mRNA expression (Memczak et al., 2013).

Disruption of circRNA–miRNA–mRNA regulatory axes has been implicated in tumor initiation, progression, metastasis, and therapeutic resistance (Memczak et al., 2013). Beyond functioning as noncoding regulators, accumulating evidence indicates that a subset of circRNAs contains internal ribosome entry sites (IRES) or m6A-driven translation initiation elements, enabling cap-independent translation into functional peptides (Fukuchi et al., 2026). These circRNA-encoded peptides have been shown to participate in signaling transduction, protein stability regulation, and feedback control of oncogenic pathways (Fukuchi et al., 2026). Therefore, both the dysregulation of circRNA-mediated ceRNA networks and the functional exploitation of circRNA-derived peptides represent critical mechanisms contributing to cancer development and provide important conceptual frameworks for understanding the interplay between RNA modification and circRNA biology (He et al., 2017; Ren et al., 2022). A growing body of evidence indicates that circRNAs play critical roles in lung cancer initiation, progression, metastasis, and therapeutic response (Yuan et al., 2025; Ren et al., 2022). Importantly, the functions of circRNAs are not solely determined by their sequences. m6A modification has been shown to significantly influence circRNA biogenesis, stability, nuclear-cytoplasmic transport, and translational potential (Xia et al., 2023). For instance, m6A can modulate circRNA expression by affecting back-splicing events; m6A readers such as YTHDF2 and IGF2BP family proteins can regulate circRNA degradation or stabilization; additionally, m6A modification can promote circRNA translation, generating biologically active peptides (Chen et al., 2019). These findings suggest that the m6A–circRNA axis may represent an important regulatory layer in tumorigenesis. In the field of lung cancer, research on m6A-modified circRNAs has been rapidly expanding, revealing their roles in tumor growth and metastasis, ferroptosis, cancer stem cell maintenance, resistance to radiotherapy/chemotherapy and targeted therapy, metabolic reprogramming, and immune microenvironment remodeling (Xie et al., 2022; Li et al., 2021). Some m6A-circRNAs have been reported to modulate oncogenic pathways via miRNA–mRNA networks, thereby promoting tumor progression or drug resistance (Wang D. et al., 2024; Zhou J. et al., 2024); others act as molecular scaffolds to facilitate protein complex formation, regulating signaling pathways or protein degradation (Li et al., 2021; Zhao et al., 2024). Moreover, m6A-circRNAs can also influence immune checkpoint expression and immune cell functions, contributing to immune escape and affecting immunotherapy responses (Chen et al., 2025; Liu Z. et al., 2021).

Based on the above background, this mini-review systematically summarizes the current research on m6A-modified circRNAs in lung cancer. We focus on six major aspects of their functional mechanisms: (1) tumor growth and metastasis; (2) ferroptosis regulation; (3) cancer stem cell maintenance and associated resistance; (4) resistance to radiotherapy, chemotherapy, and targeted therapy; (5) metabolic reprogramming; and (6) tumor immune microenvironment and immune escape. Finally, we discuss potential therapeutic strategies targeting the m6A-circRNA axis, including modulation of m6A regulators, disruption of key circRNA–miRNA–mRNA networks, and exploitation of circRNA-encoded peptides. We anticipate that these insights may provide novel theoretical foundations and directions for precision treatment of lung cancer.

## 2 Crosstalk between m6A modification and circRNAs

### 2.1 m6A regulates circRNA biogenesis, stability, and translation

**FIGURE 1:**
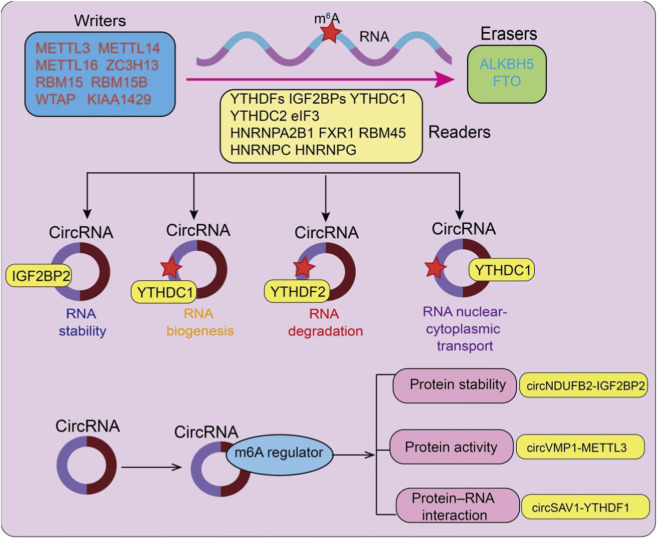
Overview of m^6^A modification and its regulatory roles in circRNA metabolism and function. m^6^A marks on circRNAs are dynamically installed by m6A “writers” (METTL3, METTL14, METTL16, WTAP, ZC3H13, RBM15/15B, and KIAA1429), removed by “erasers” (FTO and ALKBH5), and recognized by diverse “readers,” including YTH domain family proteins (YTHDF1/2/3, YTHDC1/2), IGF2BP proteins, hnRNPs, eIF3, and other RNA-binding proteins. Through these regulators, m6A modification influences circRNA biogenesis, stability, degradation, splicing, nuclear–cytoplasmic transport, and translation. In addition, circRNAs can modulate the expression or activity of m6A regulators, thereby affecting downstream processes such as protein stability, protein activity, protein–RNA interactions, and epigenetic regulation, forming complex regulatory feedback loops.

m6A modification participates in multi-layered regulation of circRNAs by influencing RNA structure and protein–RNA interactions (Chen et al., 2025; Liang L. et al., 2022). First, m6A can promote or inhibit back-splicing of pre-mRNA, thereby modulating circRNA biogenesis (Zhao et al., 2017; Wu et al., 2023) (Figure 1). Second, m6A reader proteins, such as YTHDF2 and the IGF2BP2, can bind to m6A-modified circRNAs and regulate their stability and degradation (Frye et al., 2018; Huang et al., 2024; Shi et al., 2024) (Figure 1). Finally, m6A can act as a translation initiation signal, enabling certain circRNAs to be translated into functional peptides in a cap-independent manner (Frye et al., 2018; Zhou Y. et al., 2024). This expands the biological repertoire of circRNAs beyond their conventional non-coding roles.

### 2.2 circRNAs regulate the stability and activity of m6A machinery

circRNAs are not merely downstream targets of m6A modification; they can also feedback to regulate the m6A regulatory system (Frye et al., 2018). Some circRNAs modulate the expression or activity of m6A writers (e.g., METTL3, METTL14) and erasers (e.g., FTO, ALKBH5) by sponging miRNAs or directly interacting with these enzymes (Fu et al., 2014; Liu N. et al., 2024) (Figure 1). Consequently, they can indirectly alter global or local m6A levels. In addition, circRNAs can bind to m6A reader proteins and influence their stability, subcellular localization, or RNA-binding affinity, thereby affecting m6A-mediated RNA fate decisions (Li et al., 2021). Beyond regulating circRNA biogenesis, stability, and translation, m6A modification also plays a critical role in controlling circRNA nuclear–cytoplasmic transport. Emerging evidence indicates that m6A modification facilitates circRNA export from the nucleus to the cytoplasm (Dattilo et al., 2023). CircVMP1 promotes cisplatin resistance in NSCLC by sponging miR-524-5p to upregulate METTL3, thereby enhancing malignant phenotypes and transmitting chemoresistance via exosomal transfer (Xie et al., 2022).

### 2.3 circRNAs act as scaffolds to facilitate m6A readers binding to target mRNAs

A subset of circRNAs functions as molecular scaffolds, simultaneously interacting with m6A reader proteins and their target mRNAs, thereby strengthening their interaction (He and He, 2021). CircRNAs can recruit YTHDF1 or IGF2BP family proteins to specific mRNAs, enhancing mRNA stability or translation (Jiang et al., 2022; Ling et al., 2025) (Figure 1). For example, m6A-modified circSAV1 promotes COPD progression by recruiting YTHDF1 to enhance IREB2 mRNA translation, leading to iron overload–induced ferroptosis in lung epithelial cells (Xia et al., 2023). Conversely, some circRNAs may compete with target mRNAs for binding to m6A readers, thereby blocking reader–mRNA interaction and reducing mRNA degradation or translational repression (Yuan et al., 2025). These mechanisms highlight that circRNAs are not merely “modified substrates” but also serve as critical regulatory platforms within the m6A network, fine-tuning gene expression.

## 3 Functional roles and mechanisms of m6A–circRNA crosstalk in lung cancer

**FIGURE 2:**
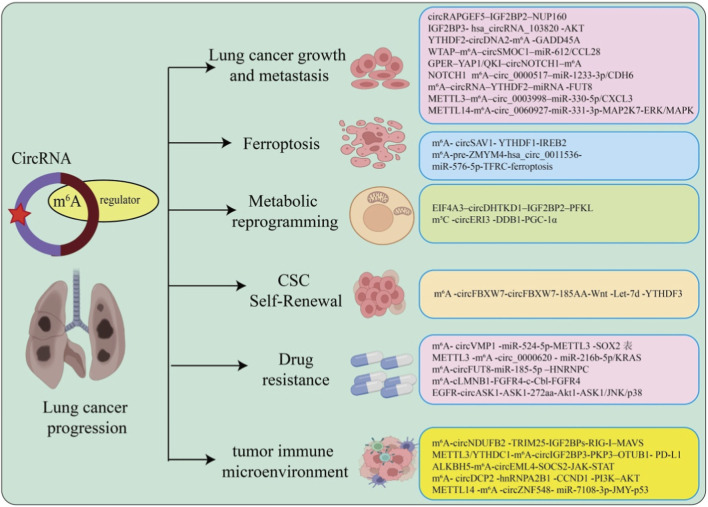
Role of m^6^A-modified circRNAs in lung cancer progression. This schematic summarizes the major functional roles of m^6^A-modified circular RNAs (circRNAs) in lung cancer. m^6^A regulators (writers, erasers, and readers) modulate circRNA stability, translation, and interactions with RNA-binding proteins or miRNAs, thereby influencing key tumor behaviors. Specifically, m^6^A-circRNAs contribute to lung cancer growth and metastasis, ferroptosis regulation, metabolic reprogramming, cancer stem cell (CSC) self-renewal, drug resistance, and the tumor immune microenvironment. Representative m^6^A-circRNA regulatory axes reported in lung cancer are listed in each functional category.

Emerging evidence indicates that m^6^A-modified circRNAs play critical roles in lung cancer progression, ferroptosis, therapy resistance, metabolic reprogramming, and immune modulation. To facilitate reader understanding, Table 1 summarizes representative m^6^A-modified circRNAs in lung cancer, including their regulatory mechanisms and functional outcomes. To illustrate the multifaceted roles of m^6^A-modified circRNAs in lung cancer, we summarize the major regulatory pathways and functional outcomes in Figure 2.

**TABLE 1:** Representative m^6^A-modified circRNAs in lung cancer and their mechanisms and functional outcomes.

circRNA	m^6^A regulator	Mechanism	Functional outcome	Reference
circRAPGEF5	IGF2BP2	Stabilizes NUP160 mRNA, inhibits autophagy	Promotes proliferation, migration, invasion, metastasis	Ling et al. (2025)
hsa_circRNA_103820	IGF2BP3	Encodes peptide that inhibits AKT pathway	Reduces viability, migration, invasion; promotes apoptosis	Zhou et al. (2024a)
circDNA2	YTHDF2	Promotes LLPS-mediated GADD45A mRNA degradation	Enhances tumorigenesis, prognostic biomarker in PM2.5-related lung cancer	Xu et al. (2025)
circSMOC1	WTAP	Sponges miR-612 → upregulates CCL28	Promotes proliferation, invasion	Zhu et al. (2024)
circNOTCH1	METTL14	Reduces m6A of NOTCH1 mRNA → increases stability	Activates NOTCH1 pathway, promotes growth	Shen et al. (2021)
circFUT8	YTHDF2	Sponges YTHDF2 and miR-186-5p → stabilizes FUT8 mRNA	Promotes malignancy	Dong et al. (2023a)
circ_0003998	METTL3	Sponges miR-330-5p → upregulates CXCL3	Promotes proliferation, angiogenesis, migration, invasion	Liu et al. (2025)
hsa_circ_0072309	FTO	Sponges miR-607 → upregulates FTO	Promotes proliferation, migration, invasion	Mo et al. (2021)
circ_0060927	METTL14	Sponges miR-331-3p → activates MAP2K7/ERK-MAPK	Promotes proliferation, migration; inhibits apoptosis	Yin et al. (2024)
circSAV1	YTHDF1	Enhances translation of IREB2 → disrupts iron homeostasis	Induces ferroptosis	Xia et al. (2023)
circVMP1	METTL3	Sponges miR-524-5p → upregulates METTL3 and SOX2	Promotes proliferation, invasion, stemness, cisplatin resistance	Xie et al. (2022)
circ_0000620	METTL3	Sponges miR-216b-5p → upregulates KRAS	Promotes proliferation, migration, cisplatin resistance	Li et al. (2024)
cLMNB1	METTL3/YTHDF2	Scaffold for FGFR4 degradation	Reverses osimertinib resistance	Qian et al. (2025)
circASK1	YTHDF2	Translated into ASK1-272aa peptide → activates ASK1/JNK/p38	Induces apoptosis, reverses gefitinib resistance	Wang et al. (2021)
circDHTKD1	IGF2BP2	Stabilizes PFKL mRNA → enhances glycolysis	Promotes proliferation, tumor growth, metabolic reprogramming	Liu et al. (2024b)
circNDUFB2	IGF2BP2	Scaffold for TRIM25–IGF2BPs complex	Inhibits tumor growth and metastasis, activates RIG-I–MAVS immune pathway	Li et al. (2021)
circIGF2BP3	METTL3/YTHDC1	Sponges miR-328-3p/miR-3173-5p → upregulates PKP3 → inhibits PD-L1 degradation	Mediates immune evasion, reduces anti-PD-1 efficacy	Liu et al. (2021a)
circEML4	ALKBH5	Increases SOCS2 m6A → activates JAK–STAT	Promotes malignancy, metastasis in NSCLC	Cheng et al. (2023)
circDCP2	hnRNPA2B1	Upregulates CCND1 → activates PI3K–AKT, promotes M2-TAM polarization	Drives pollution-related carcinogenesis, immunosuppressive microenvironment	Qin et al. (2025)
circZNF548	METTL14	Regulates exosomal miR-7108-3p → JMY-p53 activation	Enhances CD8^+^ T cell cytotoxicity, inhibits proliferation and migration	Zhao et al. (2025a)

### 3.1 m6A–circRNA crosstalk regulates lung cancer growth and metastasis

Accumulating evidence indicates that m6A-modified circRNAs contribute to lung cancer progression through diverse mechanisms, including regulation of RNA stability, modulation of signaling pathways, and interaction with m6A readers or miRNAs (Yuan et al., 2025). For example, circRAPGEF5 is upregulated in lung adenocarcinoma and binds the m6A reader IGF2BP2, stabilizing NUP160 mRNA and suppressing autophagy, thereby promoting proliferation, migration, and invasion (Ling et al., 2025) (Figure 2). In contrast, hsa_circRNA_103820 acts as a tumor suppressor by encoding a peptide that inhibits AKT signaling, reducing cell viability, migration, and invasion while promoting apoptosis (Zhou J. et al., 2024). Other oncogenic circRNAs include circDNA2, which enhances YTHDF2-mediated degradation of GADD45A via liquid–liquid phase separation, promoting tumorigenesis in pollution-induced models (Xu et al., 2025); circSMOC1, which sponges miR-612 to upregulate CCL28, enhancing proliferation and invasion in NSCLC (Zhu et al., 2024); and circNOTCH1, which reduces m6A modification of NOTCH1 mRNA, activating the NOTCH1 pathway and promoting growth (Lin et al., 2025). Similarly, circ_0000517, circFUT8, circ_0003998, hsa_circ_0072309, and circ_0060927 act via miRNA sponging and modulation of m6A readers or signaling pathways, collectively driving NSCLC progression, EMT, and metastasis (Lin et al., 2025; Dong G. et al., 2023; Liu et al., 2025; Mo et al., 2021; Yin et al., 2024).

### 3.2 Crosstalk between m6A modification and circRNA in regulating ferroptosis in lung cancer

Ferroptosis is a form of regulated cell death characterized by iron-dependent lipid peroxidation, which has been increasingly recognized as a crucial mechanism in cancer development and therapy (Zhou Q. et al., 2024). In lung cancer, dysregulation of iron metabolism and lipid peroxidation contributes to tumor progression and treatment resistance, making ferroptosis a promising therapeutic target. Emerging evidence indicates that m6A modifications and circRNAs cooperatively regulate ferroptosis in lung cancer. METTL3-mediated m6A methylation can inhibit ferroptosis by stabilizing key effectors such as SLC7A11 and activating the PTEN/PI3K/AKT pathway (Wu et al., 2025), while m6A readers like YTHDF1 and YTHDC1 further modulate ferroptosis-related targets to influence tumor progression (Yuan et al., 2023). In parallel, m6A-modified non-coding RNAs, including lncRNAs and miRNAs, also participate in ferroptosis regulation by interacting with RNA-binding proteins or downstream effectors such as GPX4 and LCN2 (Ma et al., 2024; Wang et al., 2023). Although direct evidence of circRNAs in this context is still limited, it is plausible that circRNAs act as m6A-regulated sponges or scaffolds, integrating these epigenetic signals to fine-tune ferroptosis and contribute to lung tumor growth and therapy resistance. Collectively, these findings highlight a complex network in which m6A-circRNA crosstalk may represent a novel layer of ferroptosis regulation and a potential therapeutic target in lung cancer. m6A-modified circSAV1 is markedly upregulated in COPD lung tissues. It recruits the m6A reader protein YTHDF1 to enhance the translation of IREB2 mRNA, thereby disrupting iron homeostasis and promoting lipid peroxidation (Xia et al., 2023). As a result, ferroptosis is induced in lung epithelial cells, contributing to COPD progression. *In vivo* studies further confirm that targeting circSAV1 or alleviating iron overload significantly mitigates smoke-induced ferroptosis and lung tissue damage (Xia et al., 2023). In NSCLC cells, hsa_circ_0011536 is downregulated, and its biogenesis is facilitated by m6A-mediated back-splicing of pre-ZMYM4 (Huang et al., 2025). Mechanistically, hsa_circ_0011536 functions as a sponge for hsa-miR-576-5p, thereby releasing TFRC from miRNA-mediated repression. This leads to increased TFRC expression and induction of ferroptosis, ultimately suppressing NSCLC cell proliferation (Huang et al., 2025). In summary, these findings highlight the important role of m6A–circRNA interactions in ferroptosis regulation, suggesting that targeting the m6A–circRNA–miRNA–TFRC axis may provide a novel strategy for inducing ferroptosis in lung cancer therapy.

### 3.3 Crosstalk between m6A modification and circRNA in regulating NSCLC- CSCs

CSCs are a small but critical subpopulation within tumors that drive self-renewal, metastasis, and therapeutic resistance, making them pivotal targets in lung cancer treatment (Nassar and Blanpain, 2016). In osimertinib-resistant lung adenocarcinoma stem cells, circFBXW7 is significantly downregulated, and its low expression is closely associated with alternative Wnt pathway activation, CSC self-renewal, and drug resistance. Mechanistically, m6A modification promotes the translation of circFBXW7 into the circFBXW7-185AA peptide (Li et al., 2023). This peptide interacts with β-catenin and induces its ubiquitin-mediated degradation, thereby inhibiting canonical Wnt signaling, weakening stemness, and reversing TKI resistance. Meanwhile, Wnt signaling suppresses Let-7d, thereby releasing YTHDF3 to enhance m6A-dependent translation, forming a positive feedback loop (Li et al., 2023). These findings reveal a novel m6A–circRNA peptide axis that regulates drug resistance and CSC maintenance in lung adenocarcinoma. Overall, the m6A-mediated regulation of circRNA translation and the resulting peptide-driven feedback loop highlight a critical mechanism sustaining CSC traits and TKI resistance in lung cancer.

### 3.4 Crosstalk between m6A modification and circRNA in chemo-radiotherapy resistance

m6A-modified circRNAs also contribute to therapy resistance in lung cancer. For instance, circVMP1 promotes proliferation, invasion, stemness, and cisplatin resistance via sponging miR-524-5p and upregulating METTL3 and SOX2, and exosome-mediated transfer enhances resistance in sensitive cells (Xie et al., 2022). Circ_0000620 and circFUT8 regulate chemoresistance through miRNA sponging and stabilization of KRAS and HNRNPC, respectively (Wang et al., 2025; Qian et al., 2025). In targeted therapy, circASK1 is translated into ASK1-272aa peptide, activating the ASK1/JNK/p38 pathway to reverse gefitinib resistance, while m6A-mediated degradation by YTHDF2 reduces its expression in resistant cells (Wang et al., 2021). Likewise, METTL3/YTHDF2-mediated reduction of cLMNB1 enhances sensitivity to osimertinib, highlighting the therapeutic potential of manipulating m6A–circRNA pathways (Wang et al., 2021). Collectively, these findings demonstrate that m6A-regulated circRNAs modulate tumor progression and therapy resistance through overlapping mechanisms, including miRNA sponging, modulation of m6A readers/writers, and peptide-mediated signaling, providing multiple potential targets for therapeutic intervention.

### 3.5 Crosstalk between m6A modification and circRNA in metabolic reprogramming

Metabolic reprogramming is a hallmark of cancer, and m6A-modified circRNAs have emerged as important regulators of metabolic pathways in lung cancer (Mao et al., 2024). m6A writers such as METTL3 and METTL14 enhance the stability and translation of key glycolytic and ferroptosis-related transcripts, including DLGAP1-AS2, ENO1, and PKM2, often through recruitment of m6A readers like YTHDF1 (Ma et al., 2022). This regulation promotes aerobic glycolysis, supports stemness, and inhibits ferroptosis via GPX4 activation, contributing to tumor progression (Zhang et al., 2022), metastasis, and therapy resistance. Additionally, epigenetic modulators like SETD5 facilitate m6A-dependent metabolic reprogramming, further linking histone methylation to ferroptosis regulation. These findings underscore m6A-mediated metabolic and ferroptotic networks as potential targets for therapeutic intervention and prognostic assessment in NSCLC and other malignancies. circDHTKD1 is significantly upregulated in NSCLC and possesses a stable circular structure, with its upregulation promoted by EIF4A3 (Liu Z. et al., 2024). Functionally, circDHTKD1 enhances tumor cell proliferation, tumor growth, and glycolysis. It acts as a scaffold to strengthen the interaction between PFKL and the m6A reader IGF2BP2, thereby stabilizing PFKL mRNA, increasing glycolytic activity, and driving NSCLC progression (Liu Z. et al., 2024). Hsa_circ_0003176 suppresses DDP-resistant NSCLC by promoting autophagy and inhibiting glycolysis, a metabolic shift that reverses chemoresistance. METTL3-mediated m6A modification downregulates hsa_circ_0003176, highlighting the METTL3/m6A/hsa_circ_0003176/RPS6KB1 axis as a key regulator of NSCLC metabolism and therapy response (Guo et al., 2026). Beyond m6A, m5C modification regulates circERI3, which is highly expressed in lung cancer tissues and associated with clinical progression. NSUN4-mediated m5C modification promotes nuclear export of circERI3 and enhances its function. circERI3 regulates DDB1 ubiquitination and stability, thereby promoting PGC-1α transcription, reprogramming mitochondrial function and energy metabolism, and ultimately driving lung cancer progression (Wu et al., 2024). This indicates that the m5C–circRNA–energy metabolism axis may serve as a new therapeutic target in lung cancer. Overall, m6A- and m5C-modified circRNAs orchestrate metabolic rewiring in lung cancer through RNA stability regulation and scaffolding functions, providing novel avenues for targeting tumor metabolism.

### 3.6 Crosstalk between m6A modification and circRNA in the tumor immune microenvironment

The tumor immune microenvironment critically influences lung cancer progression and response to immunotherapy, and m6A-modified circRNAs have been shown to modulate immune regulation through multiple pathways (Park et al., 2023). circNDUFB2 is significantly downregulated in NSCLC and exerts tumor-suppressive effects (Li et al., 2021). Its m6A modification enhances its scaffold function, promoting the formation of a complex between TRIM25 and IGF2BPs, thereby inducing ubiquitin-mediated degradation of IGF2BPs and inhibiting tumor growth and metastasis. Meanwhile, circNDUFB2 activates the RIG-I–MAVS innate immune pathway, promoting immune cell recruitment, revealing a novel mechanism by which m6A-circRNA cooperatively regulates protein homeostasis and TIME to suppress lung cancer progression (Li et al., 2021). m6A-modified (Liu Z. et al., 2021) is aberrantly upregulated in NSCLC and mediates tumor immune evasion. Its formation depends on the METTL3/YTHDC1 axis. circIGF2BP3 sponges miR-328-3p and miR-3173-5p to upregulate PKP3 expression. Further mechanisms indicate that the circIGF2BP3–PKP3–OTUB1 axis inhibits PD-L1 ubiquitination and degradation, weakening CD8^+^ T cell-mediated antitumor immunity and reducing the efficacy of anti-PD-1 therapy, revealing a new mechanism of immune checkpoint regulation by m6A-circRNA (Liu Z. et al., 2021). Smoking promotes NSCLC progression by inducing accumulation of M2 tumor-associated macrophages (M2-TAMs) and secretion of exosomes. Specifically, circEML4 in exosomes from CSE-induced M2-TAMs is transferred to NSCLC cells, where it binds ALKBH5 and reduces its nuclear localization, leading to increased m6A modification. This change activates the JAK-STAT pathway by regulating m6A modification of SOCS2, enhancing tumor malignancy and metastasis (Liu Z. et al., 2021). This mechanism also suggests that EVs-circEML4 may serve as a potential diagnostic biomarker for smoking-related NSCLC and highlights the key role of m6A-circRNA in the TIME and tumor progression (Liu Z. et al., 2021). Long-term exposure to environmental carbon black nanoparticles (CBNP) induces significant upregulation of circDCP2 during lung carcinogenesis (Qin et al., 2025). Its m6A modification promotes binding between circDCP2 and hnRNPA2B1, thereby upregulating CCND1 transcription and activating the PI3K–AKT pathway, driving malignant transformation.

In addition, circDCP2 promotes M2 polarization of tumor-associated macrophages through the IGF2BP3–JAK–STAT axis, forming an immunosuppressive microenvironment and accelerating lung cancer development (Qin et al., 2025). This suggests that m6A-circRNA plays a key role in pollution-related lung carcinogenesis and has potential diagnostic and therapeutic value. circZNF548 is downregulated in NSCLC tissues, and its expression is associated with better prognosis (Zhao Y. L. et al., 2025). Overexpression of circZNF548 inhibits tumor cell proliferation and migration. Mechanistically, METTL14-mediated m6A modification reduces circZNF548 levels. circZNF548 regulates exosomal miR-7108-3p, relieving suppression of the JMY-p53 pathway and enhancing CD3^+^CD8^+^ T cell cytotoxicity (increasing CD107a, IFN-γ, perforin, and granzyme B), thereby strengthening antitumor immunity and inhibiting NSCLC progression (Zhao Y. L. et al., 2025). This suggests that the m6A–circRNA–exosomal miRNA–immune axis is a potential therapeutic target.Taken together, m6A-modified circRNAs modulate the TIME through regulation of protein stability, immune checkpoint expression, and immune cell reprogramming, offering promising targets for immunotherapy and precision treatment in lung cancer.

## 4 Clinical significance of the crosstalk between m6A modification and circRNA

### 4.1 m6A–circRNA crosstalk as diagnostic biomarkers

The interplay between m6A modification and circRNA exhibits high tissue specificity and disease relevance across various cancers, highlighting its potential as a diagnostic biomarker (Chen et al., 2022; Zhao L. et al., 2025). Firstly, m6A-modified circRNAs often display significant differential expression in tumor tissues as well as in circulating blood and exosomes, and their high stability and resistance to nuclease degradation make them suitable candidates for liquid biopsy (Chen et al., 2022; Xu et al., 2024). Secondly, the m6A modification status (e.g., increased or decreased m6A levels) may reflect tumor initiation, progression, staging, or prognosis (Fu et al., 2021). Although our study identified distinct m6A-modified circRNAs and their associated mRNAs in colorectal cancer (CRC) and breast cancer subtypes, the evaluation of their clinical utility remains preliminary (Liang W. et al., 2022). While we characterized 113 circRNA–mRNA pairs consistently downregulated in CRC and identified nine candidate prognostic genes (ABCD3, ABHD6, GAB1, MIER1, MYOCD, PDE8A, RPS6KA5, TPM1, and WDR78), the diagnostic and prognostic performance of these m6A-circRNA signatures has not yet been directly compared with established clinical markers, such as EGFR mutations in CRC or hormone receptor/HER2 status in breast cancer y (Liang W. et al., 2022). Similarly, TNBC-specific circRNAs, including those derived from ZBTB16, DOCK1, METTL8, and VAV3, showed promising diagnostic accuracy (AUC >0.80) and survival correlations, but their robustness across large independent cohorts and their additive value relative to standard clinical markers remain to be validated. Future studies integrating circRNA signatures with established clinical biomarkers will be essential to determine their translational potential in precision oncology (Qattan et al., 2026). Finally, the expression profiles of m6A–circRNAs demonstrate considerable specificity, which can aid in distinguishing different lung cancer subtypes or drug resistance states (Qi et al., 2023). Therefore, m6A–circRNA signatures may provide new molecular evidence for early screening, risk stratification, and treatment monitoring (Zhang et al., 2023).

### 4.2 Therapeutic applications targeting m6A modification and circRNA in cancer

Targeting m6A modification and circRNA offers novel directions for cancer therapy. Clinically, these strategies can be implemented from two main perspectives: (1) directly modulating m6A regulators or readers: inhibiting m6A “writers” (e.g., METTL3/14) or activating “erasers” (e.g., FTO/ALKBH5) can alter the expression and function of tumor-associated circRNAs, thereby suppressing tumor growth or reversing drug resistance (Yi et al., 2025; Yankova et al., 2021; Guirguis et al., 2023; Chen et al., 2012; Su et al., 2018). (2) Targeting circRNAs as therapeutic molecules or delivery vectors: oncogenic m6A-modified circRNAs can be silenced using siRNA, antisense oligonucleotides (ASOs) (Zang et al., 2024), or CRISPR/Cas13-based approaches, while tumor-suppressive circRNAs may be restored via synthetic circRNA delivery or vector-mediated overexpression (Zhang et al., 2020; Kordyś et al., 2022). Moreover, the inherent stability and modifiable nature of circRNAs make them ideal RNA carriers for delivering therapeutic miRNAs, siRNAs, or peptide-encoding sequences, thereby expanding the potential of RNA-based therapeutics (Zhang et al., 2020; Muskan et al., 2024). While our study highlights distinct m6A-modified circRNAs with potential diagnostic and prognostic relevance in CRC and breast cancer subtypes, several translational challenges remain. Preclinical efficacy of targeting these circRNAs or modulating m6A regulators has not yet been systematically evaluated, and potential delivery barriers—including off-target effects of m6A inhibitors and the lack of optimized circRNA delivery vehicles—may limit clinical applicability (Dong J. et al., 2023). Furthermore, no ongoing clinical trials currently assess these circRNA-based or m6A-targeted interventions, underscoring the need for rigorous *in vivo* validation and development of safe and efficient delivery systems. Addressing these issues will be critical for advancing m6A-circRNA signatures from biomarker discovery to practical therapeutic applications. Recent advances in circRNA-targeted therapeutics have demonstrated promising preclinical efficacy, yet clinical translation remains in its early stages. For example, circFAM53B, specifically overexpressed in tumor cells, encodes a cryptic peptide (FAM53B-219aa) that serves as a potential tumor vaccine antigen (NCT07245901). Preclinical studies have validated its immunogenicity, leading to the development of a lipid nanoparticle (LNP)-formulated mRNA vaccine currently being evaluated in an investigator-initiated phase 1/2 trial in combination with anti-PD-1 therapy for advanced solid tumors (NCT07245901). Despite these promising results, several challenges remain, including optimization of circRNA delivery vehicles, avoidance of off-target effects from m6A modulators, and efficient tissue-specific uptake. Ongoing clinical studies, such as dendritic cell vaccines loaded with circRNA-derived cryptic peptides (NCT06530082) and first-in-human trials of circRNA-based therapeutic platforms (e.g., EP102, NCT07163325), highlight both the translational potential and the hurdles that must be addressed, including safety, dosing, and delivery efficacy. Collectively, these findings underscore the need for continued preclinical validation, delivery system refinement, and rigorous clinical evaluation to realize circRNA-based therapies as viable precision oncology interventions. In summary, the m6A–circRNA interaction network not only deepens our understanding of tumorigenesis but also provides novel molecular tools and therapeutic strategies for cancer diagnosis, classification, treatment evaluation, and personalized therapy.

### 4.3 Delivery challenges of circRNA-Targeted therapies in cancer

Despite the promising potential of circRNAs as therapeutic targets in cancer, their clinical translation faces significant delivery challenges. CircRNAs are relatively large and negatively charged molecules with limited intrinsic ability to cross cell membranes (You et al., 2025). Moreover, circulating nucleases and immune clearance mechanisms can reduce their stability and bioavailability *in vivo* (Wang et al., 2026). To address these issues, various delivery strategies have been explored, including lipid nanoparticles, viral vectors, and chemically modified circRNAs, aiming to enhance cellular uptake, stability, and tissue specificity. However, optimizing delivery efficiency while minimizing immunogenicity and off-target effects remains a major hurdle, and further research is needed to develop safe and effective circRNA-based therapies.

CircRNA-based therapies hold considerable promise due to the unique stability, covalently closed structure, and diverse regulatory functions of circRNAs, including miRNA sponging, protein scaffolding, transcriptional modulation, and peptide translation. However, effective clinical translation is currently limited by substantial delivery challenges. Efficient and targeted delivery of circRNAs or circRNA-modulating agents remains difficult, with potential obstacles including off-target effects, immune recognition, and limited tissue-specific uptake. Recent studies have emphasized the importance of optimizing delivery vehicles, such as lipid nanoparticles, extracellular vesicles, or engineered cellular platforms, to improve stability, biodistribution, and therapeutic efficacy (Guo et al., 2025). Moreover, standardization of circRNA production, degradation control, and intracellular trafficking is essential to maximize their translational potential. Addressing these challenges will be critical for realizing circRNAs as robust biomarkers and therapeutic agents in precision oncology, including applications in cancer immunotherapy, targeted therapies, and RNA-based vaccines (Ding et al., 2026).

Exosomal delivery of circRNAs offers a promising approach for cancer therapy due to their high stability, efficient cellular uptake, and prolonged expression (Liu J. et al., 2021). Natural circRNAs carried by exosomes, such as circRNA_100284 and circ-0051443, have demonstrated tumor-suppressive effects by sponging oncogenic miRNAs (Dai et al., 2018; Chen et al., 2020). However, exogenous circRNA faces challenges including low circularization efficiency and difficulty loading large molecules into exosomes. Engineered strategies, such as lentiviral vectors combined with RVG-Lamp2b-modified exosomes, enable efficient cyclization and targeted delivery of circRNAs (e.g., circRNA_DYM, circRNA_SCMH1) to specific tissues, achieving functional gene modulation in preclinical models (Song et al., 2020; He et al., 2022). These advances establish exosome-mediated circRNA delivery as a versatile and effective platform for therapeutic applications.

## 5 Conclusion and future perspectives

m6A dynamically modulates circRNA biogenesis, stability, and translation, while circRNAs reciprocally regulate the m6A machinery and act as scaffolds to fine-tune gene expression (Qin et al., 2021). Functionally, this interplay governs key cancer hallmarks, including tumor growth, metastasis, ferroptosis, stemness, therapy resistance, metabolic reprogramming, and immune microenvironment remodeling (Du et al., 2022). Clinically, m6A-modified circRNAs hold great promise as diagnostic biomarkers due to their stability, tissue specificity, and reflection of disease states (Xu et al., 2024). Furthermore, targeting the m6A–circRNA axis offers innovative therapeutic avenues, either by modulating m6A regulators or by manipulating circRNAs directly through RNA-based interventions (Androsavich, 2024). Overall, elucidating the m6A–circRNA network not only enhances our understanding of lung cancer pathogenesis but also provides a foundation for precision diagnostics and tailored therapeutic strategies.

Firstly, the regulatory modes by which m6A affects circRNAs are highly diverse. m6A can influence circRNA biogenesis, stability, subcellular localization, and translational potential, while different m6A writers, erasers, and readers may exert distinct or even opposing effects depending on the cell type or physiological and pathological context. For instance, m6A writers may promote back-splicing and circRNA generation in some cases, whereas in others they may suppress circRNA expression. Similarly, m6A readers can either stabilize circRNAs or facilitate their degradation, exhibiting bidirectional regulatory features. Such multilayered regulation suggests that the m6A–circRNA network is not a simple linear pathway, but rather a highly dynamic regulatory system.

Secondly, the functional outcomes of m6A-mediated circRNA regulation vary markedly across different cancers and even among subtypes of the same cancer, which is closely linked to tumor heterogeneity. The same m6A–circRNA axis may act as an oncogenic driver in one tumor type while functioning as a tumor suppressor in another. This divergence may be attributed to differences in genetic mutation backgrounds, microenvironmental contexts, expression profiles of m6A regulators, and downstream target networks. Therefore, future studies should integrate tumor subtypes, staging, and treatment status for refined analysis to elucidate the specific roles of m6A–circRNA interactions under distinct pathological conditions.

Thirdly, m6A modification can promote the translation of circRNA-encoded peptides or microproteins, providing a new dimension for circRNA function. Increasing evidence indicates that some circRNAs possess translational potential, and their encoded peptides play key roles in tumor cell proliferation, apoptosis, migration, and drug resistance. As a translational initiation mark, m6A may facilitate peptide production by recruiting translation initiation complexes or altering circRNA structures to enhance ribosome recognition. This field is still in its infancy, and more systematic studies are needed to validate the biological significance and clinical relevance of m6A-dependent circRNA translation.

Finally, beyond m6A, it remains an important unresolved question whether other RNA modifications, such as m5C and ac4C, similarly regulate circRNA expression and function. Existing studies have shown that m5C and ac4C modifications significantly influence mRNA stability, translation efficiency, and nuclear export; however, their roles in circRNA biology are relatively underexplored. Future multi-omics analyses may uncover a more comprehensive “circRNA epitranscriptome” and elucidate how different modifications interact to shape tumor biology. Overall, the m6A–circRNA interaction network offers a novel perspective for cancer research, but its complexity suggests that higher-throughput and more refined research strategies—combining single-cell sequencing, structural biology, and clinical validation—are required to translate discoveries into precise diagnostic and therapeutic approaches.
